# Assessment of Knowledge, Attitude, Practice, and Associated Factors of Voluntary Blood Donation in Selected Towns of Awi Zone, Injibara, Ethiopia

**DOI:** 10.1155/2024/6069684

**Published:** 2024-06-19

**Authors:** Alemu Tsega, Destaw Mullualem, Belsti Atnkut Tadesse

**Affiliations:** ^1^Department of Biology, Genetics, Injibara University, Injibara, Ethiopia; ^2^Department of Biology, Microbiology, Injibara University, Injibara, Ethiopia

**Keywords:** associated factors, attitude, knowledge, practice, voluntary blood donation

## Abstract

**Background:** Blood donation is the process of collecting blood from donors who are at low risk for infection and are unlikely to jeopardize their health by blood donation. It is a lifesaving practice for people who have lost ample volumes of blood as a result of accidents, obstetric and gynecological bleeding, severe anemia, and cancer.

**Aim:** This study is aimed at assessing knowledge, attitude, practice, and associated factors toward voluntary blood donation in Chagni, Dangila, Injibara, and Jawi towns.

**Subject and Methods:** A community-based cross-sectional study design and multistage sampling technique were employed. The data was analyzed using SPSS version 26. Both descriptive statistics and the multivariate logistic regression model were employed to determine the significance. The association between blood donation knowledge, attitude, practice, and sociodemographic variables was tested using multivariate logistic regression.

**Results:** In Chagni, 110 (55.6%), Dangila, 162 (79.0%), Injibara, 139 (73.5%), and Jawi, 165 (64.5%), towns had adequate knowledge regarding voluntary blood donation. In Chagni, 141 (74.6%), Dangila, 170 (66.4%), Injibara, 168 (82.0%), and Jawi, 148 (74.7%), towns had an adequate attitude regarding voluntary blood donation. In Chagni, 28 (14.1%), Dangila (15.3%), Injibara (29.3%), and Jawi (12.3%), towns practiced voluntary blood donation. Respondents' sex, education level, and age were found to be significantly associated with knowledge, attitude, and practice of blood donation. Those who had a degree and above were more likely (AOR = 9.239, 5.789, 5.468, and 9.72 at 95% CI) to know about blood donation relative to those who could not read and write in Chagni, Dangila, Injibara, and Jawi, respectively.

**Conclusion:** The majority of respondents had adequate knowledge and attitudes toward blood donation but had lower practices toward voluntary blood donation.

## 1. Introduction

Blood donation is a major vital component of the human body, and safe blood transfusion is essential for improving healthcare and preventing the spread of infectious diseases [1]. The World Health Organization's (WHO) recommendations suggest that 100% of people should freely donate blood to maintain a steady supply of blood [2]. Furthermore, approximately 60% of blood is obtained from replacement or paid donors in underdeveloped nations, where over 80% of the world's population resides. The government must provide a sufficient, safe supply of blood, blood products, and services to fulfill the needs of all patients in a timely, economical, and effective way, even though blood transfusion is frequently assigned to a nongovernmental organization [3]. Ethiopia has a high rate of motor vehicle accidents, high maternal mortality (676 per 100,000), and a sizablenon-immunenimmune malaria population. Ethiopia needs 100,000 units of blood per year; however, only 43% of that amount is donated. Ethiopia has the lowest percentage of voluntary blood donors (VBDs) among the WHO's African member states (22%), which is incredibly low [4].

Individuals who donate blood under duress or in exchange for money are less likely to disclose issues that would disqualify them as donors; as such, they pose a potentially higher danger to the security of the blood supply. Donors of blood who voluntarily give their blood do so without any financial or other type of compensation [5]. Ethiopia is grouped as one of the countries with a very low blood donation rate, which is 0.6 per thousand population, next to Nigeria [6]. The National Blood Bank Service of Ethiopia and its network currently provide safe blood and blood products to 52% of medical facilities [7]. A study conducted in nations in sub-Saharan Africa, including Ethiopia, found a strong correlation between maternal mortality and the inability to obtain blood transfusions. Specifically, the study found that maternal hemorrhage deaths accounted for 26% (16%–72%) of all maternal deaths. With an estimated population of over 110 million, Ethiopia is the second most populous country in Africa. It ranks among the top 10 countries in the world for motor accidents, has a high rate of nonimmune malaria cases, and most medical care requires a steady supply of blood because one in seven hospital patients requires it [8]. The Ethiopian blood bank has been gathering about 200,000 units of blood from donors a year in previous years. Nonetheless, the nation needs about 18,000 units of blood every day, but only about 1100 units are typically collected, leaving 16,900 units short of what is needed [9]. An adequate and safe blood supply has proven to be difficult to come by in Ethiopia, a country marked by significant disparities in access to blood. Only 1100 units of blood are being collected daily in Ethiopia, despite the country's 18,000 unit requirement [10]. Patients who need specific blood products or components during a blood crisis include pregnant women, cancer patients, and those suffering from anemia-causing illnesses. These patients can have critical transfusion demands. Individuals with severe injuries from auto accidents may suffer significant morbidity and death from anemia when there is an excessive demand for blood [11]. One major issue that still exists in the Awi zone of several municipalities is the inadequate supply of blood. Lack of will, low community understanding of the value of voluntary nonremunerated blood donation, societal stigmas and misunderstandings, restricted service infrastructure growth, insufficient staff, restricted donor counseling, and restricted media access are the main obstacles to advancement [12]. Barriers to blood donation include medical reasons, fear (needles, dizziness, etc.), lifestyle barriers, lack of marketing communication, lack of knowledge about blood donation, and negative experiences related to blood donation [11]. One of the biggest risks to blood services during and after a COVID-19 outbreak is a decrease in donor numbers. This study's main goal was to evaluate community-based voluntary blood donation practices, knowledge, attitudes, and related factors in a few chosen towns in Ethiopia's Amhara regional state, Awi zone.

## 2. Materials and Methods

### 2.1. Description of the Study Area

**Figure 1:**
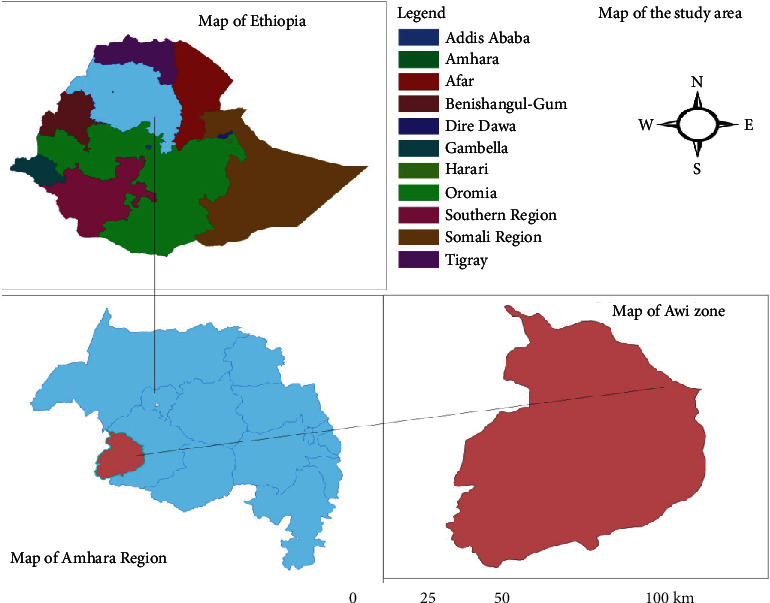
Map of the study area created by the author.

Amhara regional state has 12 zones, and the Awi zone is where this study was carried out. Injibara is the capital city of the Awi zone (Figure 1). Its coordinates are as follows: latitude: 10°23′ N to 10°85′ N; longitude: 36°35′ E to 36°57′ E; altitude: 1800–3100 m above sea level. In addition, there are 46 health centers and 4 hospitals, totaling 1339 active healthcare providers [13].

### 2.2. Study Design and Period

A community-based cross-sectional quantitative survey was carried out between June and October 2022/2023.

### 2.3. Sample Size

All of the adult inhabitants who had spent at least 6 months living in the chosen towns of the Awi zone were the sources of the population. This study includes residents who were within the 18–65 age range. The following assumption was used to determine the sample using the single population proportion formula: Due to the lack of published data regarding blood donation knowledge, attitudes, and practices in this specific study area, 50% of the sample size was selected with a 95% confidence interval, a 10% marginal error (*d*), and a design effect of 2. The sample size was determined using the formula below, which was devised by Cochran (1963, 1975) to produce a representative sample for the proportions of large samples [14].

 $$\begin{array}{c} n=\frac{Z^{2}\left(\alpha /2\right)*}{d^{2}}p\left(1-p\right)=384\text{ of respondents} \end{array}$$

where *z*^2^ *α*/2 = 1.96 at a 95% confidence interval, *P* is the prevalence of voluntary blood donation which is equal to 50% because there is no previous study, and *d* = 10% which is a tolerable error between the sample and the true population. The overall sample size was 848 respondents for three kebeles, with a 5% marginal error and a design effect of 2.

Therefore, the sample size of each kebele in a town was calculated by using proportional allocation as follows with respect to the number of respondents:

 $$\begin{array}{c} n=\frac{n*Ni}{N} \end{array}$$

The sample size for each Kebele was allocated in the following formula:

 $$\begin{array}{c} N\overset{N}{\underset{k=0}{\sum }}\text{N}\text{i},\overset{n}{\underset{k=0}{\sum }}\text{n}\text{i} \end{array}$$

### 2.4. Sampling Design

Participants in the study were recruited through a multistage sampling process. Using a straightforward random sampling technique, four towns were chosen from the 10 districts and four administrative towns in the Awi zone for the first sampling stage. The participants selected from each household were selected to participate in the study. Using a straightforward random sampling technique, four kebeles were chosen at random from among all the kebeles in each town for the second sample phase. To choose homes from each of the kebeles, a systematic sampling procedure was used. Proportionate-to-population size analysis was used to establish the number of households sampled from kebeles and the chosen municipalities [15]. The study comprised 848 people in total, all of whom were residents of towns for at least 6 months and ranged in age from 18 to 65. The study excluded participants with a history of medical conditions or treatments that postpone permanent or temporary blood donation, as well as those who had recently undergone blood transfusions [16]. Blood donation knowledge was graded as “1” for accurate responses and “0” for inaccurate ones. Individuals who correctly answered more than 50% of the knowledge assessment questions were deemed to have adequate knowledge regarding blood donation, whereas those who scored less than 50% were deemed to have inadequate knowledge. In a similar view, questions about attitudes were given a score of “1” for accurate answers and “0” for inaccurate answers. Individuals who correctly answered more than 50% of the questions about attitude were deemed to have a positive attitude about blood donation, whereas those who scored less than 50% were deemed to have a poor attitude. Practice: People were deemed to have practiced if they had donated blood at least once throughout their lives. Some people had never donated blood before [17].

### 2.5. Data Collection

Relevant data were gathered using a self-designed, pretested, tightly structured, open-ended, and closed-ended questionnaire. A structured, self-administered questionnaire that was standardized was used to gather primary data from personnel. The survey was written in English, translated into Amharic, and then back into English by a second translator to ensure uniformity. Three sections made up the questionnaire: eight knowledge questions, ten attitude questions, seven practice questions about blood donation, and sociodemographic characteristics (age, sex, marital status, religion, educational status, and occupation). Sixteen individuals (data collectors) were chosen to serve as data collectors for particular kebeles of a town [17].

### 2.6. Data Quality Control

The data collection method was pretested on 2% of the sample size of participants in each selected kebele which was out of the study sample. Furthermore, 2-day training was given to data collectors. During the data collection period, the supervisors supervised the data collectors frequently, and the collected data was checked for consistency and completeness on a daily basis. In the case of a quality problem, it was brought up for discussion, and necessary corrections were made by returning the questionnaire to the respective data collector. After data collection, the collected data were entered into Excel by different investigators and checked for consistency.

### 2.7. Data Analysis

SPSS version 26 was the statistical program used to examine the data. Before doing bivariate and multivariate logistic regression analyses using inferential statistics, descriptive statistics were generated, and the findings were displayed in tables and percentages. Using multivariate logistic regression analysis, the relationship between the dependent, including knowledge, attitude, and practice of voluntary blood donation, and explanatory factors was investigated. To find variables independently linked with blood donation, all bivariate analysis variables with a *p* value < 0.1 were added to the final multiple logistic regression models. To identify variables that were independently linked to blood donation, multivariable logistic regression was used. The adjusted odds ratio (AOR) was shown to be significant at *p* value < 0.05 of confidence intervals concerning blood donation knowledge, attitude, and practice [18].

## 3. Results

### 3.1. Chagni Town's Demographic Characteristics (*N* = 198)

Out of the 198 respondents, 64 (32.3%) were women and 134 (67.7%) were men. A higher percentage of participants (*n* = 89, or 44.9%) were between the ages of 36 and 45. Approximately 114 (57.6%) of the survey participants had completed their studies up to the degree level. One hundred thirty-six (68.7%) of the survey participants identified as Orthodox Christians.

### 3.2. Knowledge, Attitude, and Practice of the Study Respondents

Of the respondents, 110 (55.6%) had sufficient understanding regarding blood donation, while 88 (44.4%) had inadequate information. A total of 148 respondents (74.7%) had positive attitudes toward blood donation, and voluntary donation was thought to be the most effective way to obtain donors. While the majority of respondents had never donated blood in their lives, 28 research participants (14.1%) had done so more than once.

### 3.3. Factors Linked to Blood Donation Knowledge in Chagni Town (*K* = 198)

A multivariate logistic regression model revealed a significant relationship between the respondent's age, sex, educational attainment, and understanding of blood donation. The likelihood of knowledge was higher among respondents with a degree or above (AOR = 9.239, 95% CI: 2.458–61.788). The likelihood that a respondent between the ages of 36 and 45 knew about blood donation was higher (AOR = 1.197, 95% CI: 1.060–6.41). Male respondents had higher knowledge (AOR = 17.171, 95% CI: 4.916–59.979).

### 3.4. Factors Associated With Attitude of Blood Donation

Age, sex, education level, and religion were found to be strongly correlated with attitudes toward voluntary blood donation in multivariate logistic regression analysis. AOR = 41.293, 95% CI: 1.454–172.680, showed that respondents with a degree or above were 41.3 times more likely to have a positive attitude about blood donation. Muslims were 0.37 times less likely to have an attitude against blood donation (AOR = 0.37, 95% CI: 2.561–9.577) than non-Muslim respondents (Table 1).

### 3.5. Factors Associated With Practice of Blood Donation

The study found a substantial correlation between the education level, religion, sex, and age of respondents in multivariate logistic regression analysis and their practice of blood donation. The age group of 36–45 respondents had a higher likelihood of donating blood (AOR = 3.506, 95% CI: 11.400–30.710). Compared to female participants, males had a greater likelihood of donating blood (AOR = 1.153, 95% CI: 1.019–1.254). Furthermore, respondents with a degree or higher in education had a higher likelihood of donating blood than nonrespondents (AOR = 5.016, 95% CI: 11.000–32.125) (Table 2).

### 3.6. Dangila Town Demographic Characteristics (*N* = 205)

One hundred twenty-one (59.0%) of the 205 participants were men, and 84 (41.0%) were women. A greater proportion of the participants (*n* = 94, or 45.9%) fell between the ages of 36 and 45. Of the survey participants, 83 (40.9%) had completed their education to a degree or higher.

### 3.7. The Respondents' Knowledge, Attitudes, and Practices to the Study

Of the respondents, 162 (79.0%) had a sufficient understanding of blood donation, while 43 (21.0%) had inadequate knowledge. Eighty-six (82.0%) of the respondents had a positive attitude toward blood donation, and voluntary donation was regarded as the most effective way to obtain donors. While 15 research participants (7.3%) have made many donations, the majority of respondents had never given blood in their lives.

### 3.8. Factors Linked to Blood Donation Knowledge

The results of the multiple logistic regression model indicated a significant relationship between the respondent's knowledge of blood donation and their sex, religion, and educational attainment. Knowledge was greater among study participants with degrees and above than among students (AOR = 5.789, 95% CI: 17.48–29.591). Age groups between 36 and 45 were associated with higher odds of blood donation knowledge (AOR = 1.732, 95% CI: 1.223–2.402).

### 3.9. Factors Associated With Attitude of Blood Donation

Age, sex, education level, and religion were found to be substantially correlated with attitudes toward voluntary blood donation in the multivariate logistic regression model. In comparison to those who cannot read or write, individuals with a degree or above were more likely (AOR = 11.043, 95% CI: 23.78–54.734) to have a positive attitude about blood donation. In comparison to female respondents, male respondents were more likely (AOR = 2.466, 95% CI: 4.920–6.611) to have a positive attitude about blood donation (Table 3).

### 3.10. Factors Associated With Practice of Blood Donation

Age, education level, and respondent sex were all strongly correlated with the research area's voluntary blood donation practices in multivariate logistic regression analysis. When compared to government employees, those with a degree or higher had a higher likelihood of donating blood (AOR = 7.321, 95% CI: 1.056–50.772). Compared to those who cannot read and write, males were more likely to donate blood (AOR = 5.34, 95% CI: 23.45–67.2110). Additionally, the respondents in the age range of 36–45 had a higher likelihood of donating blood than the other respondents (AOR = 34.274, 95% CI: 1.046–21.633) (Table 4).

### 3.11. Blood Donation in Injibara Town in Relation to Independent Variables

One hundred thirty-three (52.0%) of the 189 participants were men, and 123 (48.0%) were women. A greater proportion of participants (*K* = 107, 41.8%, and 105, 41.0%) fell into the 36–45 and 18–35 year age ranges, respectively. About 177 (69.1%) of the participants were identified as followers of an Orthodox faith in relation to voluntary blood donation.

### 3.12. The Respondents' Knowledge, Attitudes, and Practices to the Study

Of the responders, 165 (64.5%) had sufficient information about donating blood, while 91 (35.5%) had inadequate understanding about donating blood voluntarily. A total of 170 (66.4%) respondents agreed that voluntary blood donation was the best way to obtain donors, and they had a positive attitude about it. Of the study participants, 29 individuals (11.3%) had made multiple blood donations; yet, this was the majority.

### 3.13. Factors Linked to Blood Donation Knowledge

The multivariate logistic regression model's outcome indicated that the respondent's age group, sex, occupation, degree of education, and religion were all substantially correlated with their knowledge of blood donation.

Study participants with a degree or more had a higher likelihood of being aware of blood donation (AOR = 5.468, 95% CI: 23.578–34.90). Ages 36–45 were the study's most likely respondent group to know about blood donation (AOR = 1.392, 95% CI: 1.692–2.798).

### 3.14. Factors Related to Blood Donation Attitude

Age, sex, religion, and education level were all strongly correlated with attitudes toward blood donation in multivariate logistic regression. Higher degree holders were more likely to have a positive attitude about blood donation (AOR = 6.23, 95% CI: 23.89–56.293). Respondents who identified as Protestants were less likely to donate blood voluntarily (Table 5).

### 3.15. Factors Associated With Practice of Blood Donation

According to multivariate logistic regression analysis, respondents' age, sex, education level, and employment status were all strongly correlated with their blood donation practices. Private sector employees had a higher likelihood of donating blood (AOR = 1.216, 95% CI: 3.368–4.367). Compared to female participants, males had a greater likelihood of donating blood (AOR = 7.3529, 95% CI: 7.3529 (5.136–25.914)). Conversely, respondents with a degree had a higher likelihood of donating blood (AOR = 6.267 (23.412–98.574)) (Table 6).

### 3.16. Jawi Town Respondents for Independent Variables

Of the 189 participants, 41 (21.7%) were female and 148 (78.3%) were male. A greater proportion of participants (*n* = 129, or 63.8%) fell between the ages of 36 and 45. Of the survey participants, about 62 (32.8%) had completed a degree-level program. Among the survey participants, 124 (65.6%) adhered to the Orthodox religion, while the remaining 57 (30.2%) were Muslims.

### 3.17. Knowledge, Attitude, and Practice of the Study Respondents

Of the respondents, 139 (73.5%) had sufficient understanding regarding voluntary blood donation, while 50 (26.5%) had inadequate information. A total of 141 respondents (74.6%) had positive attitudes toward blood donation, and voluntary donation was thought to be the most effective way to find donors. Twelve research participants (6.3%) had given blood more than once in their lives, while the majority of respondents had never given blood at all.

### 3.18. Factors Associated With Knowledge of Blood Donation

The multivariate logistic regression model's outcome indicated that the respondent's age, sex, religion, and educational attainment were all substantially correlated with their knowledge of blood donation.

Those with a degree or more were more likely to be informed study participants (AOR = 9.72, 95% CI: 43.17–92.851). Participants in the study who were 36–45 years old had a lower likelihood of knowing about blood donation (AOR = 0.028, 95% CI: 0.003–0.264).

### 3.19. Factors Associated With Attitude of Blood Donation

Age, sex, and education level were found to be strongly correlated with attitudes toward blood donation in multivariate logistic regression. In comparison to those who cannot read and write, those with degrees were 45.75 times more likely (AOR = 45.75, 95% CI: 34.6–78.210) to have a positive attitude about blood donation (Table 7).

### 3.20. Factors Associated With Practice of Blood Donation

#### 3.20.1. Multivariate Analysis

Participant sex, educational attainment, religion, employment position, and age were all substantially correlated with blood donation practices in multivariate logistic regression analysis. The likelihood of blood donation was eight times lower for those participants who worked in the commercial sector (AOR = 0.418, 95% CI: 32.367–75.941). In terms of blood donation, male participants were more likely than female participants to donate blood 79 times (AOR = 79.00, 95% CI: 21.10–73.430). The likelihood of blood donation was higher among respondents with a degree or higher than among others (5.65; AOR = 5.65, 95% CI: 18.60–72.59) (Table 8).

## 4. Discussion

In order to better understand respondents' knowledge, attitudes, and practices about blood donation in the towns of Chagni, Dangila, Jawi, and Injibara, the researchers attempted to look into these topics in this study. In all, 110 (55.6%), 162 (79.0%), 165 (64.5%), and 139 (73.5%) of the study participants knew enough about blood donation in the towns of Chagni, Dangila, Jawi, and Injibara, respectively. The purpose of this community-based cross-sectional study, which was carried out in a chosen town, was to evaluate the degree of voluntary blood donation among 845 participants in terms of knowledge, attitude, and practice, as well as related factors. However, this result is in line with a study carried out in Debre Marko's town, Northwest Ethiopia, that sought to evaluate the general level of knowledge, attitude, and practice about blood donation as well as related factors [19]. Multivariate logistic regression in the current study revealed that the variables that were substantially connected with the participants' knowledge were sex, education level, occupation, and education. In Chagni, Dangila, Jawi, and Injibara towns, respectively, participants with a degree or above (AOR = 9.239, 95% CI: 2.458–3461.788), (AOR = 5.789, 95% CI: 17.48–29.591), (AOR = 5.468, 95% CI: 23.578–34.90), and (AOR = 9.72, 95% CI: 43.17–92.851) were more likely to have adequate knowledge regarding blood donation. Therefore, participants' awareness of blood donation rises along with their level of education. The majority of research participants (66.4%–84%) have positive attitudes toward blood donation. This study is similar to another that was carried out in the town of Gondar, Northwest Ethiopia, where the majority of the 630 participants (82%) had a high attitude toward voluntary blood donation [15]. One probable explanation for this disparity could be the variations in the sociocultural elements. Using multivariate logistic regression, the only variable in this study that was substantially correlated with the participants' attitudes was education. Multivariate logistic regression in this study revealed that the variables that were substantially correlated with blood donation knowledge, attitude, and practice were sex, education, and age group. In each municipality included in this study, 134 (67.7%), 121 (59.0), 133 (52.0), and 148 (78.3%) of the donors were men. The outcome is consistent with research carried out in the town of Gondar, which revealed that 94 blood donors, or 66.6% of the total, were men. Compared to females, men were more likely to donate blood (AOR = 1.153 (1.019–1.254), 12.121 (2.022–6.72), and 7.3529 (0.136–0.914) [20]. This discrepancy could be caused by the educational attainment and medical experience of the donor regarding blood donation. Blood donation rates were higher among respondents with a degree or above (3.247; AOR = 3.247, 95% CI: 1.408–7.489) than among other respondents. Regarding the practice of voluntary blood donation, in Chagni, 28 (14.1%), Dangila (15.3%), Injibara (29.3%), and Jawi (12.3%) were volunteers, which is comparable with the study conducted in Bale. However, this result is higher for voluntary blood donation practice in Birbir town (16.8% voluntary practice) but less than in Bahir Dar (63.7%) in the study. The increment in this study could be due to the awareness-creation and mobilization activities done at Injibara University in collaboration with Injibara Comprehensive Hospital.

In the meantime, this study has demonstrated an inverse association between age and motivation to contribute in terms of practice level. That is, compared to older people, younger people might be more motivated to donate. This is consistent with a study that found age influences blood donation behavior. In a similar vein, research has indicated that elderly adults are less likely to donate blood. A plausible explanation for this could be that the younger age group consists of university students who may possess adequate knowledge about the blood donation procedure, perhaps resulting in a favorable attitude toward blood donation [21]. There were only three kebeles included in the study sample. Therefore, the results cannot be applied to participants from other towns or to other kebeles in the Awi zone. The accuracy of the participants' reporting is the only factor that ensures the dependability of our data. Thus, recollection bias may manifest. Additionally, as indicated by the pretest, only the most prevalent incentives and obstacles to voluntary blood donation have been investigated.

## 5. Conclusion

Overall, this study demonstrated that a significant percentage of participants had sufficient knowledge about voluntary blood donation and exhibited a positive attitude toward voluntary blood donation. Nonetheless, the level of blood donation practice was low since the main reasons people did not donate blood were fear of becoming anemic after giving blood and concern about health risks. Knowledge of blood donation was found to be substantially correlated with educational status. Religion was the only variable that continued to be substantially correlated with attitudes toward factors influencing blood donation. In addition to age, other statistically significant factors influencing blood donation practices across all municipalities included sex and religion. The predictor variables for voluntary blood donation among respondents in each location were age, willingness to contribute in the future, fear of donating blood, willingness to encourage relatives to donate, and attitude toward voluntary blood donation. Town residents should endeavor to raise responders' awareness of voluntary blood donation through cooperation with the local blood bank. Concentrating on the predictor variables found will recommend having adequate blood in the blood bank, if not in the zone where voluntary blood donation is practiced. Thus, it is suggested that regularly scheduled awareness creation and voluntary blood donation campaigns be organized at the community level to utilize potential donors who lack the practice of donating blood. We advise upcoming researchers to carry out highly analytical investigations to evaluate the voluntary blood donation process.

## Figures and Tables

**Table 1:** Multivariate analysis of sociodemographic variable and attitude about blood donation among respondents living in Chagni town (*n* = 198).

**Variables**	**Categories**	**p** **value**	**AOR (95% CI)**
Age group	18–35	0.001	1
Age group (1)	36–45	0.041	1.535 (1.516–4.565)^∗^
Age group (2)	46–65	0.001	0.054 (0.011–0.262)^∗^
Age group (3)	> 65	0.426	0.385 (0.037–4.041)
Sex (1)	Male	0.044	2.193 (1.765–6.283)^∗^
Religion	Orthodox	0.506	1
Religion (1)	Muslim	0.046	0.37 (2.561–9.577)^∗^
Religion (2)	Protestant	0.044	0.061 (2.145–7.760)^∗^
Education level	Cannot read and write	0.004	1
Education level (1)	1–8 attended	0.020	1.290 (3.008–10.574)^∗^
Education level (2)	9–12 attended	0.04	1.391 (67.357–429.842)^∗^
Education level (3)	Certificated	0.011	3.335 (2.937–35.901)^∗^
Education level (4)	Diploma	0.030	9.396 (1.763–132.979)^∗^
Education level (5)	Degree and above	0.029	41.293 (1.454–172.680)^∗^
Occupation	Governmental employee	0.247	1
Occupation (1)	Student	0.448	0.465 (0.064–3.359)
Occupation (2)	Housewife	0.561	0.936 (0.078–110.940)
Occupation (3)	Private worker	0.41	0.030 (1.114–9.56)
Occupation (4)	Unemployed	0.472	0.337 (0.018–6.503)
Marital status	Single	0.004	1
Marital status (1)	Married	0.001	8.706 (2.333–32.485)^∗^
Marital status (2)	Divorced	0.883	0.898 (0.217–3.726)

*Note:* 1 = reference.

Abbreviations: AOR = adjusted odds ratio, CI = confidence interval.

^*^ *p* value < 0.05.

**Table 2:** Multivariate analysis of sociodemographic variables and practice about blood donation among respondents living in Chagni town (*n* = 198).

**Variables**	**Categories**	**p** **value**	**AOR (95% CI)**
Age	18–35	0.021	1
Age (1)	36–45	0.017	3.506 (11.400–30.710)^∗^
Age (2)	46–65	0.024	0.386 (1.058–2.561)^∗^
Age (3)	> 65	0.078	44.696 (0.652–3063.328)
Sex (1)	Male	0.040	1.153 (1.019–1.254)^∗^
Religion	Orthodox	0.181	1
Religion (1)	Muslim	0.007	0.109 (18.722–51.704)^∗^
Religion (2)	Protestant	0.020	0.320 (19.360–30.661)^∗^
Education level	Cannot read and write	0.132	1
Education level (1)	1–8 attended	0.959	1.117 (0.017–72.587)
Education level (2)	9–12 attended	0.804	1.734 (0.023–132.288)
Education level (3)	Certificated	0.036	1.846 (4.67–24.896)^∗^
Education level (4)	Diploma	0.011	2.111 (7.001–21.025)^∗^
Education level (5)	Degree and above	0.007	5.016 (11.000–32.125)^∗^
Occupation	Governmental employee	0.385	1
Occupation (1)	Student	0.908	0.89 (0.67–76.98)
Occupation (2)	Housewife	0.084	0.838 (0.143–56.399)
Occupation (3)	Private worker	0.204	0.433 (0.399–73.921)
Occupation (4)	Unemployed	0.362	0.265 (0.015–4.609)
Marital status	Single	0.009	1
Marital status (1)	Married	0.22	26.029 (0.188–212.502)
Marital status (2)	Divorced	0.063	27.696 (0.838–915.445)

*Note:* 1 = reference.

Abbreviations: AOR = adjusted odds ratio, CI = confidence interval.

^*^ *p* value < 0.05.

**Table 3:** Results of a multivariate analysis of sociodemographic variables and respondents' attitudes toward blood donation in Dangila town (*n* = 205).

**Independent variables**	**Categories**	**p** **value**	**AOR (95% CI)**
Age	18–35	0.283	1
Age (1)	36–45	0.024	31.304 (4.091–11.023)^∗^
Age (2)	46–65	0.191	0.384 (0.091–1.611)
Age (3)	> 65	0.003	0.361 (7.054–12.415)^∗^
Sex (1)	Male	0.033	2.466 (4.920–6.611)^∗^
Religion	Orthodox	0.036	1
Religion (1)	Muslim	0.013	0.928 (9.580–14.790)^∗^
Religion (2)	Protestant	0.116	0.335 (0.085–1.312)
Education level	Cannot read and write	0.007	1
Education level (1)	1–8 attended	0.045	3.718 (7.972–14.227)^∗^
Education level (2)	9–12 attended	0.001	2.645 (3.516–145.825)^∗^
Education level (3)	Certificated	0.024	6.824 (1.552–6.028)^∗^
Education level (4)	Diploma	0.027	7.45 (15.960–17.341)^∗^
Education level (5)	Degree and above	0.014	11.043 (23.78–54. 734)^∗^
Occupation	Governmental employee	0.49	1
Occupation (1)	Student	0.544	0.693 (0.213–2.262)
Occupation (2)	Housewife	0.288	0.438 (0.095–2.010)
Occupation (3)	Private worker	0.938	1.052 (0.293–3.782)
Occupation (4)	Unemployed	0.245	0.414 (0.093–1.834)
Marital status	Single	0.007	1
Marital status (1)	Married	0.049	0.365 (0.134–0.994)^∗^
Marital status (2)	Divorced	0.001	0.042 (0.007–0.267)^∗^
Marital status (3)	Widowed	0.3450	95.14 (0.054–0.076)

*Note:* 1 = reference.

Abbreviations: AOR = adjusted odds ratio, CI = confidence interval.

^*^ *p* value < 0.05.

**Table 4:** Multivariate analysis of sociodemographic variables and practice of voluntary blood donation among respondents living in Dangila town (*n* = 205).

**Independent variables**	**Categories**	**p** **value**	**AOR (95% CI)**
Age	18–35	0.02	1
Age (1)	36–45	0.015	34.274 (1.046–21.633)^∗^
Age (2)	46–65	0.953	1.052 (0.196–5.651)
Age (3)	> 65	0.258	3.587 (0.393–32.752)
Sex (1)	Male	0.016	12.121 (2.022–6.72)^∗^
Religion	Orthodox	0.525	1
Religion (1)	Muslim	0.347	0.311 (0.027–3.550)
Religion (2)	Protestant	0.025	0.404 (1.044–3.740)
Education level	Cannot read and write	0.860	1
Education level (1)	1–8 attended	0.576	2.034 (0.168–24.5490)
Education level (2)	9–12 attended	0.127	0.076 (0.032–0.067)
Education level (3)	Certificated	0.036	2.785 (2.61–29.701)^∗^
Education level (4)	Diploma	0.48	3.891 (12.60–21.321)^∗^
Education level (5)	Degree and above	0.021	5.34 (23.45–67.2130)^∗^
Occupation	Governmental employee	0.034	1
Occupation (1)	Student	0.39	1.502 (0.275–8.213)
Occupation (2)	Housewife	0.138	0.082(0.011–0.067)
Occupation (3)	Private worker	0.044	7.321 (1.056–50.772)^∗^
Occupation (4)	Unemployed	0.646	1.742 (.163–18.603)
Marital status	Single	0.042	1
Marital status (1)	Married	0.705	45.296 (0.138–653.790)^∗^
Marital status (2)	Divorced	0.16	0.275 (0.380–277.786)
Marital status (3)	Widowed	0.780	0.012(0.0560–0.089)

*Note:* 1 = reference.

Abbreviations: AOR = adjusted odds ratio, CI = confidence interval.

^*^ *p* value < 0.05.

**Table 5:** Multivariate analysis of sociodemographic variable and attitude about blood donation among respondents living in Injibara town (*n* = 256).

**Independent variables**	**Categories**	**p** **value**	**AOR (95% CI)**
Age	18–35	0.036	1
Age (1)	36–45	0.006	11.575 (23.791–43.139)^∗^
Age (2)	46–65	0.012	0.821 (2.330–20.44)^∗^
Age (3)	> 65	0.336	2.200 (0.442–10.953)
Sex (1)	Male	0.278	1.391 (0.766–2.524)
Religion	Orthodox	0.021	1
Religion (1)	Muslim	0.849	2.071 (0.527–2.179)
Religion (2)	Protestant	0.026	0.305 (0.107–0.867)^∗^
Education level	Cannot read and write	0.001	1
Education level (1)	1–8 attended	0.001	6.028 (2.427–14.971)^∗^
Education level (2)	9–12 attended	0.001	2.749 (2.855–21.031)^∗^
Education level (3)	Certificated	0.001	4.720 (1.993–11.181)^∗^
Education level (4)	Diploma	0.045	5.546 (18. 671–56.892)^∗^
Education level (5)	Degree and above	0.0023	6.23 (23.89–56.293)^∗^
Occupation	Governmental employee	0.016	1
Occupation (1)	Student	0.212	1.524 (0.696–3.337)
Occupation (2)	Housewife	0.279	1.935 (0.586–6.391)
Occupation (3)	Private worker	0.022	0.946 (2.422–12.121)^∗^
Occupation (4)	Unemployed	0.51	0.715 (0.230–2.219)
Marital status	Single	0.726	1
Marital status (1)	Married	0.003	0.985 (7.521–11.861)
Marital status (2)	Divorced	0.23	0.507 (0.150–1.708)
Marital status (3)	Widowed	0.073	21.169 (0.881–0.972)

*Note:* 1 = reference.

Abbreviations: AOR = adjusted odds ratio, CI = confidence interval.

^*^ *p* value < 0.05.

**Table 6:** Multivariate analysis of sociodemographic variables and practice about blood donation among respondents living in Injibara town (*n* = 256).

**Independent variables**	**Categories**	**p** **values**	**AOR (95% CI)**
Age	18–35	0.005	1
Age (1)	36–45	0.015	0.216 (0.062–0.746)^∗^
Age (2)	46–65	0.13	2.294 (0.776–6.783)
Age (3)	> 65	0.425	2.138 (0.331–13.795)
Sex (1)	Male	0.032	7.3529 (5.136–25.914)^∗^
Religion	Orthodox	0.028	1
Religion (1)	Muslim	0.38	0.774 (0.267–2.249)
Religion (2)	Protestant	0.510	0.554 (0.095–3.219)
Education level	Cannot read and write	0.186	1
Education level (1)	1–8 attended	0.100	4.031 (0.765–21.248)
Education level (2)	9–12 attended	0.72	1.320 (0.202–8.628)
Education level (3)	Certificated	0.044	1.929 (3.359–10.348)
Education level (4)	Diploma	0.049	3.567 (23.412–78.234)^∗^
Education level (5)	Degree and above	0.039	6.267 (23.412–98.574)^∗^
Occupation	Governmental employee	0.038	1
Occupation (1)	Student	0.044	0.429 (1.513–3.980)^∗^
Occupation (2)	Housewife	0.085	0.290 (0.017–0.086)
Occupation (3)	Private worker	0.07	1.268 (3.368–4.367)^∗^
Occupation (4)	Unemployed	0.35	0.25 (0.143–5.969)
Marital status	Single	0.244	1
Marital status (1)	Married	0.092	2.506 (0.861–7.292)
Marital status (2)	Divorced	0.48	0.491 (0.048–5.012)
Marital status (3)	Widowed	0.760	0.320 (17.98–67.432)

*Note:* 1 = reference.

Abbreviations: AOR = adjusted odds ratio, CI = confidence interval.

^*^ *p* value < 0.05.

**Table 7:** Multivariate analysis of sociodemographic variables and attitudes about blood donation among respondents living in Jawi town (*n* = 189).

**Independent variables**	**Categories**	**p** **value**	**AOR (95% CI)**
Age	18–35	0.029	1
Age (1)	36–45	0.057	2.610 (5.971–7.017)^∗^
Age (2)	46–65	0.012	1.251 (2.198–7.897)^∗^
Age (3)	> 65	0.36	2.491 (0.303–20.444)
Sex (1)	Male	0.03	2.515 (5.858–7.371)
Religion	Orthodox	0.363	1
Religion (1)	Muslim	0.786	0.18 (0.344–2.241)
Religion (2)	Protestant	0.14	0.765 (0.501–45.308)
Education level	Cannot read and write	0.077	1
Education level (1)	1–8 attended	0.003	0.956 (2.11–4.321)^∗^
Education level (2)	9–12 attended	0.013	3.319 (2.064–12.588)^∗^
Education level (3)	Certificated	0.048	11.163 (5.249–15.432)^∗^
Education level (4)	Diploma	0.021	23.87 (11.12–89.4)^∗^
Education level (5)	Degree and above	0.045	45.75 (34.6–78.210)^∗^
Occupation	Governmental employee	0.061	1
Occupation (1)	Student	0.087	2.903 (0.857–9.833)
Occupation (2)	Housewife	0.070	3.751 (0.358–39.340)
Occupation (3)	Private worker	0.024	0.579 (7.218–11.540)
Occupation (4)	Unemployed	0.353	0.484 (0.105–2.237)
Marital status	Single	0.028	1
Marital status (1)	Married	0.003	3.844 (1.602–9.224)
Marital status (2)	Divorced	0.079	1.988 (0.430–9.193)
Marital status (3)	Widowed	0.092	1.948 (0.760–87.45)

*Note:* 1 = reference.

Abbreviations: AOR = adjusted odds ratio, CI = confidence interval.

^*^ *p* value < 0.05.

**Table 8:** Multivariate analysis of sociodemographic variables and practice about blood donation among respondents living in Jawi town (*n* = 189).

**Independent variables**	**Categories**	**p** **value**	**AOR (95% CI)**
Age	18–35	0.075	1
Age (1)	36–45	0.043	3.474 (2.056–4.008)^∗^
Age (2)	46–65	0.038	1.761 (18.56–110.397)^∗^
Age (3)	> 65	0.025	0.318 (1.944–23405.373)^∗^
Sex (1)	Male	0.027	79.00 (21.10–73.430)^∗^
Religion	Orthodox	0.393	1
Religion (1)	Muslim	0.041	0.114 (1.004–3.160)^∗^
Religion (2)	Protestant	0.804	0.964 (0.009–407.212)
Education level	Cannot read and write	0.145	1
Education level (1)	1–8 attended	0.031	0.985 (7.3–13.296)^∗^
Education level (2)	9–12 attended	0.022	1.051 (12.001–22.212)^∗^
Education level (3)	Certificated	0.758	31.612 (7.7–33.892)^∗^
Education level (4)	Diploma	0.011	42.45 (13.60–34.789)^∗^
Education level (5)	Degree and above	0.036	55.65 (18.60–72.59)^∗^
Occupation	Governmental employee	0.024	1
Occupation (1)	Student	0.049	0.896 (32.297–42.116)^∗^
Occupation (2)	Housewife	0.078	0.320 (0.870–0.921)
Occupation (3)	Private worker	0.039	0.418 (32.367–75.941)^∗^
Occupation (4)	Unemployed	0.099	0.6540 (0.730–0.988)
Marital status	Single	0.093	1
Marital status (1)	Married	0.55	31.371 (0.551–64.318)
Marital status (2)	Divorced	0.18	0.677 (0.691–40.498)
Marital status (3)	Widowed	0.0870	0.016 (0.170–0.690)

*Note:* 1 = reference.

Abbreviations: AOR = adjusted odds ratio, CI = confidence interval.

^*^ *p* value < 0.05.

## Data Availability

The data used in this study are available from the corresponding author on reasonable request.

## Supporting Information

Supporting Information Additional supporting information can be found online in the Supporting Information section. We collected the data and information based on the questionnaire prepared in English and translated into the native language of the respondents. We have attached the questionnaire in the supporting portion of the system.
