# Supplementary material for: Assessment of Knowledge, Attitude, Practice, and Associated Factors of Voluntary Blood Donation in Selected Towns of Awi Zone, Injibara, Ethiopia
Source: Biomed Res Int. 2024 Jun 19;2024:6069684. doi: 10.1155/2024/6069684 (PMC11458273; doi:10.1155/2024/6069684)
Supplement: Supporting Information — Additional supporting information can be found online in the Supporting Information section. We collected the data and information based on the questionnaire prepared in English and translated into the native language of the respondents. We have attached the questionnaire in the supporting portion of the system. [file 6069684.f1.docx]

**
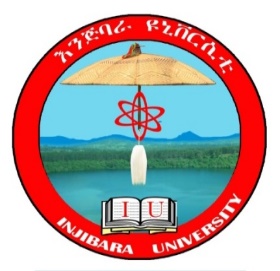
**

**INJIBARA UNIVERSITY**

**College of Natural and Computational Science**

Assessment of knowledge, attitude, practice and **associated factors of voluntary blood Donation in selected towns of** Awi zone**,** Ethiopia

One of the missions and activities of our university given by the Ministry of Education is to solve and support the problems of the local community with the help of research. Therefore, we teachers of Injbara University, in addition to teaching, are also doing problem-solving studies and research on various issues. We are also conducting an evaluation study based on the knowledge, attitude, practice and related challenges of voluntary blood donation in our community in our city. The result of this research is to continue and organize the efforts of the relevant body and the community to overcome the problems of blood shortage in our region as well as in our city. Therefore, without any doubt, we would like to ask you how to fill this written questionnaire honestly. We are deeply grateful for all your cooperation.

Note: It is not necessary to write the individual's name. Section One: Socio-Popular Information **Questionnaire Directions: - To answer the following questions, circle the letter that contains the answer you chose**

1. Age. a. 18-35 b. 36- 45 c. 64-65 d. More than 65

2. Gender a. Female b. male

3. Religion a. Orthodox b. Muslim c. Protestant d. If yes, specify ---------------

4. Education level a. cannot read and write b. 1-8 educated

c. 9-12 educated d. Certificate e. Diploma f. degree and above

5. Working condition a. Government employee b. student c. Housewife

d. Private employee

6. Marital status a. Married b. Unmarried c. A man/woman who has passed away

d. She is divorced

**Guideline 2: Answer your knowledge on blood donation.**

Directions: To answer the following questions, circle the letter that contains the answer of your choice.

1. Is donating blood harmful? a. Yes b. It is not

2. Do you know the common blood types in humans? a. Yes b. I do not

3. Do you know your blood type? a. Yes b. I does not know

4. If yes to question #3, please state your blood type. ------------------------------------

5. Where is the place to donate blood? a. Hospital b. Health center

c. Red Cross d. Donation center

6. What is the purpose of blood donation? a.Sve the life of my relative b. To save the life of a person c. Getting insurance d. Accepting payment

7. What is the minimum age to donate blood? a. 18 years old b. Under 18 years

c. Over 65 years d. I don't know

8. What is the maximum age for donating blood? a. 65 years old b. above 65 years

c. Under 65 d. Not known

9. What is the minimum weight to donate blood?

a. 45 kg below b. 45 kg c. More than 45 kg d. I does not know

10. What is the maximum amount of blood to donate at one time?

a. 250 ml. 350 ml b. 450 ml. c. I does not know

11. How often does a person think they can donate blood?

a. Every 3 months b. Every 6 months c. Once a year d. twice in three months

12. Can women donate blood during menstruation? a. Yes b. No

13. Which of the following is the best source of blood donation?

a. Voluntarily b. By replacing c. in payment d. I do not know

14. Where can you find information about blood donation? a. From the media b. From health professionals c. School d. From the Red Cross e. From literature f. If you accept

15. Can you list the diseases that can be transmitted when blood is received from an infected person?

----------------------------------------------------- -------------------------------------------------- -- -----------------------------------------------

**Guideline 3: Answer your questions about donating blood.**

Directions: To answer the following questions, circle the letter that contains the answer you chose

1. Donating blood is a moral responsibility. a. I agree b agree very much

c. I do not agree d. I strongly disagree e. I am not sure

2. Are you willing to donate blood in the future? a. Yes b. I am not

3. Turn number 2. If you say no, what is your reason? -----

4. Who encourages you to donate blood? a. Yes b.Not encouraged

5. Ordinary number 4. What is your reason if List is not encouraged? -

6. Are you willing to become a regular blood donor? a. Yes b.I am not

7. Turn number 6. If you say no, what is your reason? ----------------------------------------- -----

7. Does it say that blood should be donated only in emergencies? a. I agree very much. B.I agree

c. I strongly disagree d. I do not agree

8. People who donate blood are temporarily exhausted. a. I agree very much. I agree

c. I strongly disagree d. Disagree e. I will not

9. What do you think about donating blood? a. It's good for. They are very good

c. It is very bad d. It's bad

10. Do you think there is a need for incentives to donate blood? a. Yes b It is not necessary

11. What do you think will happen to you when or after donating blood?

a. Acquires infection b. for temporary depression c. fall d. getting sick

12. He wants to donate blood only to family members and friends.

a. I agree very much. b I agree c. I strongly disagree d. I do not agree

13. Do you want to donate blood to a guest who needs blood?

a. I agree very much. b I agree c. Disagree d. I strongly disagree

**Guideline 4: Give feedback on your experience with blood donation.**

Directions: To answer the following questions, circle the letter that contains the answer you chose

1. Did he know about blood donation before? b. Yes b. No

2. Random Number 1 If yes, how often have you participated in blood donation?

a. Once upon a time. b twice c. three times d. More than three times

3. Why you donate blood?

a. To replace b To get satisfaction c. To get paid d. To be famous

4. How do you feel after donating? a. depressed b.. Fear c. fever d. Anger

e. If yes, specify -----------------------------

5. What are your reasons for donating blood?

a. Moral responsibility b. thinking of others c. Peer pressure

d. To reduce weight e. other (specify)---------------------------

6. Why don't you donate blood?

a. Fear of needle pain b. Ineligibility to donate blood

c. Fear of being weak d. No one has ever asked me how

e. If yes, please specify------------------------------

7. What do you think the blood bank organization can do so that more people become blood donors?

a. Conducting educational campaigns about the importance of blood donation

b. Confirmation of confidentiality of donors after blood samples are tested

c. Give incentives to blood donors

d. Place ads on TV, radio, website about blood donation

e. There should be blood banks in accessible hospitals

f. If yes (specify) ---------------------------------------------- ------------------------------
